# Identifying menstrual migraine– improving the diagnostic criteria using a statistical method

**DOI:** 10.1186/s10194-019-1035-7

**Published:** 2019-09-06

**Authors:** Mathias Barra, Fredrik A. Dahl, E. Anne MacGregor, Kjersti Grøtta Vetvik

**Affiliations:** 1 The Health Services Research Unit – HØKH, Akershus University Hospital, Lørenskog, Norway; 2 C3 – Centre for Connected Care, Oslo University Hospital, Oslo, Norway; 3 Institute of Clinical Medicine, University of Oslo, Oslo, Norway; 4 Centre for Neuroscience, Surgery and Trauma, Blizard Institute of Cell and Molecular Science, Barts and the London School of Medicine and Dentistry, London, UK; 5 Centre for Reproductive Medicine, St. Bartholomew’s Hospital, London, UK; 6 Department of Neurology, Akershus University Hospital, Lørenskog, Norway

**Keywords:** Menstrually related migraine, Diagnostic criteria, Statistical criteria, Markov chain model, Operations research

## Abstract

**Objective:**

To develop a robust statistical tool for the diagnosis of menstrually related migraine.

**Background:**

The International Classification of Headache Disorders (ICHD) has diagnostic criteria for menstrual migraine within the appendix. These include the requirement for menstrual attacks to occur within a 5-day window in at least $\frac {2}{3}$ menstrual cycles ($\frac {2}{3}$-criterion). While this criterion has been shown to be sensitive, it is not specific. Yet in some circumstances, for example to establish the underlying pathophysiology of menstrual attacks, specificity is also important, to ensure that only women in whom the relationship between migraine and menstruation is more than a chance occurrence are recruited.

**Methods:**

Using a simple mathematical model, a Markov chain, to model migraine attacks we developed a statistical criterion to diagnose menstrual migraine (sMM). We then analysed a data set of migraine diaries using both the $\frac {2}{3}$-criterion and the sMM.

**Results:**

sMM was superior to the $\frac {2}{3}$-criterion for varying numbers of menstrual cycles and increased in accuracy with more cycle data. In contrast, the $\frac {2}{3}$-criterion showed maximum sensitivity only for three cycles, although specificity increased with more cycle data.

**Conclusions:**

While the ICHD $\frac {2}{3}$-criterion is a simple screening tool for menstrual migraine, the sMM provides a more specific diagnosis and can be applied irrespective of the number of menstrual cycles recorded. It is particularly useful for clinical trials of menstrual migraine where a chance association between migraine and menstruation must be excluded.

## Introduction

### Menstrual migraine

The International Classification of Headache Disorders 3 (ICHD3) provides diagnostic criteria for *menstrual migraine without aura (MM)*^1^[1]. As these criteria have not been thoroughly validated they are placed in the appendix.

The criteria are based on three main features:

1. The *type* of migraine: migraine without aura (MO);
2. The *timing* of attacks in relation to menstruation: they should occur during the *menstrual window*, i.e. the 5-days starting two days before onset of menstruation until the third day of bleeding (i.e. day 1±2); and
3. The *frequency* of attacks in relation to menstruation: attacks should be present in at least two out three consecutive menstruations.

The term MM covers two subtypes: A1.1.1 *pure menstrual migraine (PMM)*, and A1.1.2 *menstrually related migraine (MRM)*. Women with PMM have exclusively perimenstrual attacks, while women with MRM have additional non-menstrual attacks. The focus of this research is on MRM, and to some extent, PMM. Here we refer to the above ICHD-criteria jointly as the *two-out-of-three* ($\frac {2}{3}$)*-criterion* and to MRM diagnosed by this criterion as $\frac {2}{3}MRM$.

There is evidence to support features 1 and 2; i.e. the migraine type and the timing of the attacks [2–7]. However, the third feature, considering the frequency of attacks on menstrual days, is not statistically sound although it was originally introduced to rule out spurious association between menstruation and migraine [8–10]. These criticisms remain forceful even when migraine-diaries of high quality are available for the patients. A pertinent question is: how to ensure that attacks with menstruation are not occurring by chance [9]?

It is debated whether MM should be regarded as MO triggered by menstruation, or, if MM constitutes a distinct entity [9, 11, 12]. Indeed, after decades of research, the pathophysiological mechanisms of MM are poorly understood. In order to further penetrate these mechanisms it is crucial that a homogeneous population of patients – where the association between menstruation and migraines is greater than chance – is studied.

### Statistical criteria

To appreciate the problem, an inherent shortcoming with the $\frac {2}{3}$-criterion is that it is neither sensitive nor specific for a de facto association: the $\frac {2}{3}$-criterion risks including women where the association is entirely absent, [8, 10] and, conversely, the $\frac {2}{3}$-criterion may exclude women with a clear and statistically significant association. This occurs when migraine attacks are less frequent (e.g. women with migraine attacks in every second menstruation and only very rarely outside the menstrual window). Furthermore, it unclear how the criteria are to be applied to diaries with more than three 5-day menstrual windows.

Partly to address the concern regarding spurious associations, a *probability criterion (PC)* for MRM was proposed by Marcus et al. [8] Unfortunately, the PC’s original formalization was mathematically flawed. Later, Barra et al. published a corrected version of the PC, together with a simulation-analysis of its test-characteristics [10].

The statistical test that underpins the PC from Barra et al. [10] relies on a *non-clustering assumption* for correct size: the criterion’s rate of type I errors. The non-clustering assumption (or the *independence of attacks assumption*) asserts that there is a day-to-day constant and independent probability of migraine that is unaltered by observing headaches. However, this assumption does not hold. Migraine days do cluster. According to the ICHD definition migraine attacks may last up to three days (72 h) untreated or unsuccessfully treated [1]. In a recent study^2^, it was shown that about 50% of migraine attacks are expected to span more than one day [13].

The aim of the present work is to develop the PC into a more robust statistical criterion for MRM, which is independent of the clustering of attacks. By focusing on the number of migraine *attacks* – rather than the number of migraine *days* falling inside or outside the menstrual window – the simple statistical test (and its interpretation) from Barra et al. can be retained [10]. This leads to a novel and statistically attractive alternative diagnostic criterion for MM: *statistical MM (sMM)*. Furthermore, we analyse a data set of migraine diaries, to compare the $\frac {2}{3}$MRM to the sMM, and discuss differences, and their implications for further research on MM. We also assessed the new criterion’s accuracy in a simulation study.

We appreciate that the sMM criterion developed here necessitates somewhat more complicated calculation and book-keeping of the migraine diaries than the PC from Barra et al., [10] but argue that this trade-off is worthwhile. On this note, some of the materials presented over the next sections might appear intimidating to the mathematically untrained. However, the mathematics presented is quite simple, and most readers will be able to understand the formulae and reasoning with some efforts. This is not to say that it is *easy* to penetrate all the details, nor that a quick read-through will suffice for a full understanding. The “Discussion” section therefore begins with a very simplified account of what we have done.

A note on the terminology is warranted. The term MM is taken to mean menstrual migraine, and includes both the pure variant (PMM) and menstrually related migraine (MRM). In this article, $\frac {2}{3}$MRM and sMM (and the PC from Barra et al. and Marcus et al.) denote diagnostic criteria for MRM. However, the sMM criterion can diagnose PMM, since sMM will also classify most migraine diaries displaying PMM as a case of sMM. There is clearly a strong statistical association between menstruation and migraine in women with PMM, and the sMM criterion will identify this.

## Methods

### Theory

**Fig. 1:**
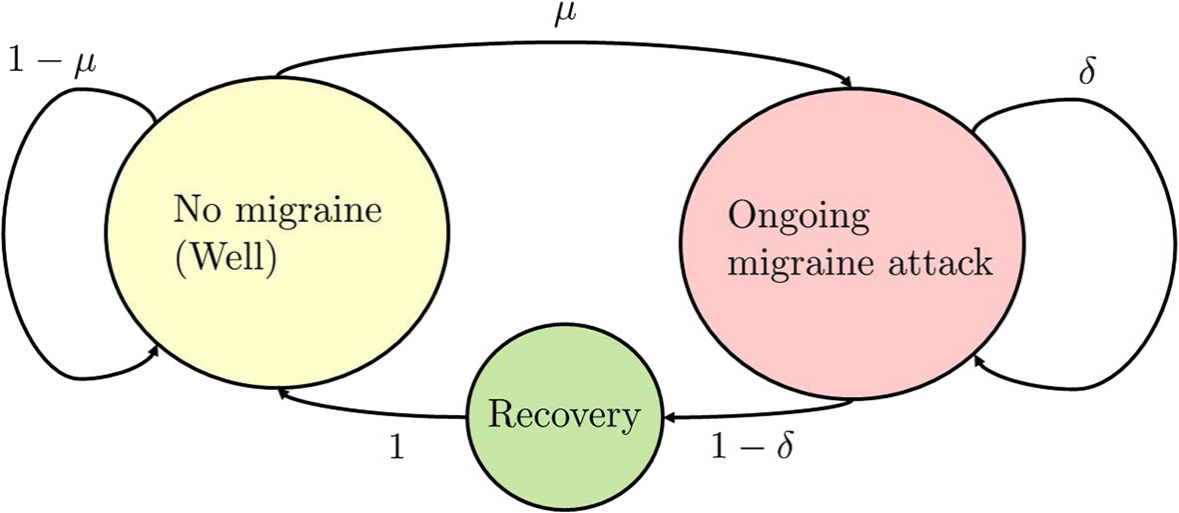
Simple Markov chain model of the progression of migraine attacks. The transition probabilities *μ* and *δ* represent the probability of onset of a migraine attack – conditioned on being susceptible – and the probability that an ongoing attack will continue, respectively. We assume that each patient may have individual transition probabilities. The non-clustering assumption excludes *μ*≠*δ* in general. MM – interpreted within this simple model – postulates that women with MM deviate from the base-model above, and instead have two distinct migraine probabilities: *μ*^NM^ which represents her probability of migraine attack-onset outside her menstrual window, and *μ*^M^ which represents an elevated migraine attack-onset probability during their menstrual window. That is, MM is present when *μ*^M^>*μ*^NM^

In this paper we will assume that migraine attacks can be modelled by the simple Markov chain model in Fig. 1, as suggested by Barra et al.[13]

Within this framework, MM can be defined as a patient’s tendency to have an increased migraine probability (*μ*^M^) during her 5-day menstrual windows, as compared to the non-menstrual migraine probability (*μ*^NM^). We may then ask: does the individual patient experience a statistically significant increase in the probability of migraine onset during the menstrual window?

The previous publications on the PC used a very simple exact test (one-sided Fisher’s Exact with mid-p correction [10, 14]) yielding *p*-values for a null-hypothesis of non-association between menstruation and migraine, so that low *p*-values indicate a likely association between menstrual window-days and migraine days (*p*-values are inherently hard to interpret; [15] we give a precise statement below). In terms of the Markov chain model, the non-clustering assumption is equivalent to *μ*≠*δ*. But, the assumption of non-clustering is empirically false: migraine days do cluster [13].

Here we show that by focusing on when attacks start – that is estimating individuals’ *μ*’s based on their headache diaries – we retain most of the simplicity, and all of the statistical rigour, of the PC, while relaxing the non-clustering assumption.

The main points about the criterion we introduce below are: that the *p*-values are computed from a patient’s 2×2-table classifying days on which a migraine attack could start, as any of the four possible combinations of menstrual vs. non-menstrual, and, migraine started vs. migraine did not start. Secondly, that a one-sided test is employed: we are only interested in patients with an elevated migraine probability during the menstrual window. A two-sided test would be unnecessarily conservative for our purposes, and furthermore obscure the desired interpretation of the resulting *p*-value. This *p*-value can be interpreted thus:

- there is *no association* between the patient’s migraine attack pattern and the menstrual cycle, so that there is *no increased probability* of observing migraine attacks on menstrual days, i.e.:
  $$\mu^{\mathrm{M}}-\mu^{\text{NM}}\stackrel{\text{\tiny def}}{=}\Delta\mu=0$$
- the probability of seeing attacks start as *frequently* within the patient’s menstrual windows, compared to outside them, as observed in the patient’s diary, equals *p*.

Hence, a ‘low *p*’ means that association between menstruation and migraine is *likely*.

**Fig. 2:**
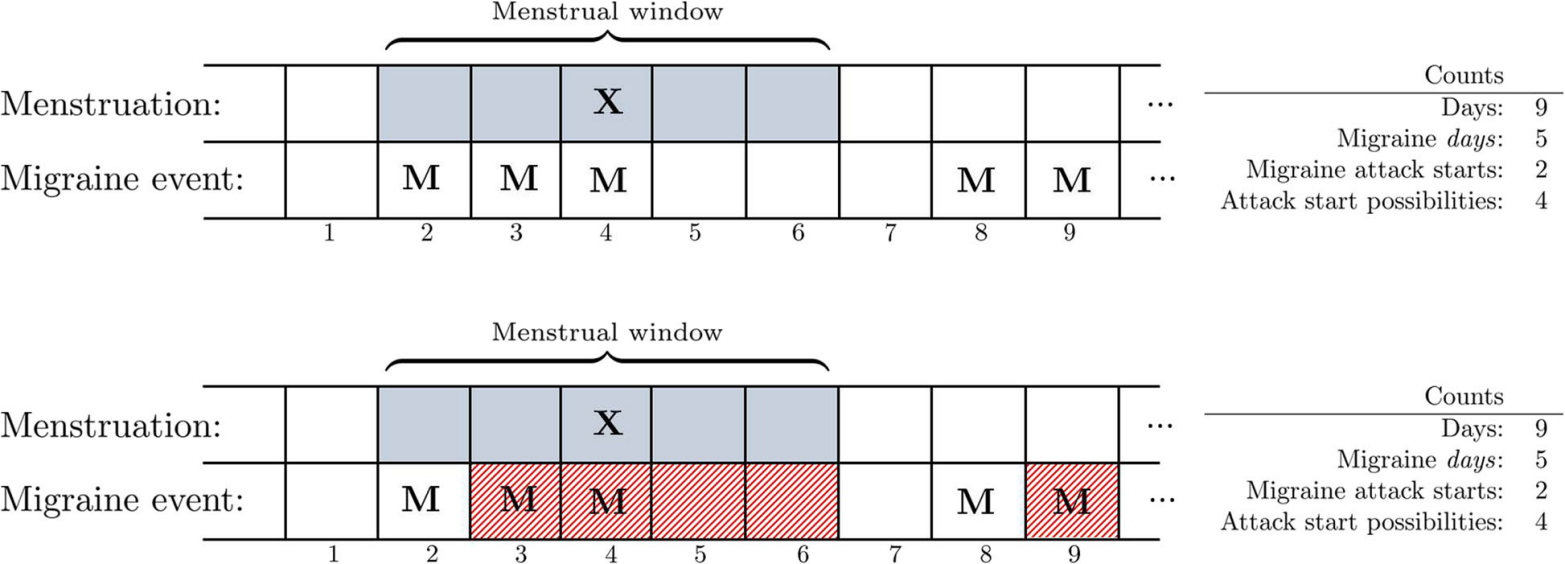
Illustration of headache diaries and how to count *migraine days* versus *migraine attack starts*

**Fig. 3:**
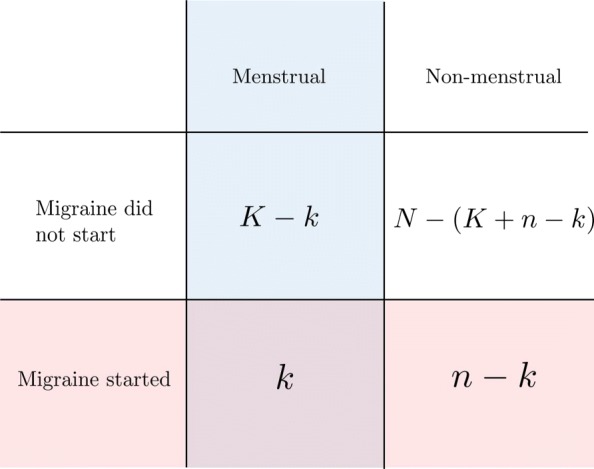
The 2×2contingency table underlying the statistical test. All days on which a migraine could start is classified as within or outside the menstrual window, and, as having had an attack start or not

Consider the excerpt from a hypothetical headache diary given in Fig. 2. The first row records a first day of a menstrual bleeding on the fourth day (**X**), meaning that the days 2—6 define a 5-day menstrual window (indicated by shading). In the second row, each day on which migraine was present is indicated (**M**), i.e. days 2—4, 8 and 9. Counting migraine days within and outside the menstrual window yields that out of the *N*=9 days (of the excerpt) we count *n*=5 migraine days in total, *k*=3 menstrual migraine days, and *K*=5 menstrual days, for the following contingency table (Fig. 3):

These key figures can then be used to compute the probability of seeing *k* (or more) migraine days falling within the menstrual window days, given that we have observed a total of *N* days, out of which *n* were migraine days by the following formulae:

1 $$ \begin{aligned} f_{\text{HG}}(k,K,n,N)= &\frac{ {K\choose k}{N-K\choose n-k}}{{N\choose n}} \end{aligned}  $$

2 $$ \begin{aligned} p(k,K,n,N) \,=\, &\underbrace{\left(\sum_{i = k}^{\min(n,K)}f_{\text{HG}}(i,K,n,N)\right)}_{P(X \geq k\vert N,K,n\geq k)} \,-\, \underbrace{\frac{1}{2}f_{\text{HG}}(k,K,n,N)}_{\text{mid-} {p} \text{ correction}} \end{aligned}  $$

Formula (1) specifies the probability mass function *f*_HG_ for the hyper-geometric distribution: it computes the probability of seeing exactly *k* migraine days within the menstrual window, given that migraine days are equally likely to occur on any day. Formula (2) gives the *p*-value we seek; the sum of the probabilities of values *i* that is greater than or equal to *k*. The last term is the mid-p correction, which is justified for our purposes because *n* itself is random prior to observing each woman’s diary. For a further discussion of this test see Lydersen et al. and Barra et al. [10, 14] However, the non-clustering assumption is crucial for this test to be of correct size. For an appropriate statistical test the size should be dominated by the pre-set significance level, so that for a significance level of e.g. *α*=0.05, the probability of rejecting non-association ought never to exceed 5% on a sample of diaries satisfying the null-hypothesis.

#### Removing the need for non-clustering – trimming: counting only *attack-starts*

Returning to the Markov chain model, we realize that it can be appropriate to perform the test just discussed if we focus solely on days of the headache diary which corresponds to the transition probability *μ*. Under the null-hypothesis, this parameter ought not to be influenced by whether or not a day falls within a menstrual window. Conversely, if *μ* depends on the menstrual status, then we could hope to detect this by the one-sided test for *Δμ*=0 versus the alternative hypothesis *Δμ*>0.

This can be achieved quite straightforwardly by subjecting the headache diaries to what we call *trimming*. Trimming is illustrated in the bottom panel of Fig. 2. Now, we ignore information from days on which an attack is ongoing, and consider only information from days on which an attack may potentially start. Note that we must also disregard any two days immediately following a migraine attack, and also make sure that only migraine attacks with an identifiable start are included.

The rationale for trimming has been explained in Barra et al. [13] as well as in the guidelines for controlled trials of drugs in migraine, in which the International Headache Society considers that any headache pain from 2—48h after initial pain freedom should be considered a relapse, i.e. *part of the same attack* [16]. As a consequence we must count so-called *migraine locked days* – i.e. days that are immediately preceded and immediately succeeded by migraine days – as a migraine day. For example, if day 3 in Fig. 2 had been recorded in the diary as a non-migraine day which was ‘migraine-locked’ by migraine days recorded on days 2 and 4, then day 3 would be imputed as a migraine day. We refer to Barra et al. for a more detailed exposition of how to map days to Markov chain states, and for a justification for imputing onto migraine-locked days.

By performing this trimming, we may classify the *remaining* diary-days according to the exact same logic before, and furthermore, revert to using the formulae (1&2, p. 23) above. Importantly, this test will have size equal to the chosen *α*-level, regardless of the behaviour of the *δ*-transitions.

Returning to Fig. 2, the days removed by trimming are 3—6 and 9 (hatched in the second row). This yields *N*=4,*n*=2,*K*=1, and *k*=1 for computing the *p*-value.^3^ We now have all the pieces necessary for our proposed *statistical MM diagnosis*:

**Statistical Menstrual Migraine – sMM(** ***α*** **)**

1. Migraine without^4^ aura;
2. A trimmed (migraine-locked free) headache diary’s one-sided Fischer Exact mid-p corrected *p*-value <*α* on a test of *Δμ*=0.

This diagnosis is properly a family of diagnoses: any *α*<0.5 defines a possible cut-off, hence e.g. sMM(0.1) means that an *α*-level of 0.1 has been employed – more on this in the empirical part of the study.

### Data

We used a data set of headache diaries from 165 women attending the City of London Migraine Clinic during the period 1998—1999; details on this data set has been published previously [4]. Importantly, none of the women were using hormonal contraception, all initial diagnoses of migraine type headache were set by headache experts, and only records with a minimum of three consecutive menstrual cycles were included in our study; other characteristics of the migraine episodes (e.g. laterality) were not relevant for the method being developed here, and were not analysed.

We computed the length (in days) of each menstrual cycle, and the individual mean cycle lengths. Cycles of duration longer than twice that woman’s (individual) mean cycle length, were assumed to represent missing data, and the respective portion of the headache diaries were omitted. For example, if a woman displayed cycle lengths of (28, 28, 80, 28, 28, 28) days, we retained only the latter three cycles in the final analysis; in the case (28, 28, 80, 28, 28) the entire diary was excluded, as three *consecutive* cycles were not extractable. We imputed migraines on any migraine-locked days. Furthermore, to ensure that no migraines were erroneously registered as within or outside a menstrual window, all diaries were truncated at 15 days prior to the first, and 15 days post the last, registered menstrual bleeding. We computed descriptive statistics (means, medians, inter-quartile ranges (IQR)) for the number of cycles, migraine days and attacks, and migraine-locked days, both for the individual women and for the pooled data.

### Diagnosing

Diagnosing the women was done by each of the two methods; the $\frac {2}{3}$MRM and the sMM. the $\frac {2}{3}$MRM diagnoses were set by an algorithm which verified that a migraine attack started within $\geq \frac {2}{3}$ of the menstrual windows. Furthermore, an sMM *p*-value was computed for each patient based on her trimmed diary.

### Analysis

We compared the sub-groups of patients diagnosed with each of the two diagnoses, considering various levels of *α* as a cut-off. Descriptive statistics were computed for each group for comparison. Empirical parameters for the Markov chain (*μ*, *μ*^NM^, *μ*^M^) were estimated from the data.

The specificity of the test is the chosen *α*-level – by construction. The sensitivity of the test depends on numerous circumstances, but clearly increases in both *Δμ*=*μ*^M^−*μ*^NM^ and the numberof days/menstrual cycles in a diary [10].

Since a true ‘gold standard’ for MM does not exist we conducted a simulation study to explore the two criterions’ test-characteristics by ROC curve analysis and AUC-scoring [17, 18]. The idea here is to exploit the Markov chain model so that we can generate two sample populations, one of true positives and one of true negatives. The Markov chain model, was populated by sampling from the empirical distributions of *μ*’s, drawing from the patients who were diagnosed with *both*$\frac {2}{3}$MRM and sMM(0.1) for simulating true positives (*μ*^M^ and *μ*^NM^), and patients receiving *neither* diagnosis for simulating true negatives (*μ*). We simulated 10 000 diaries containing three menstrual windows for 28-day cycles (23 + 5 days) together with 10 days into the fourth cycle, for each category. Each diary was diagnosed for sMM(0.1) and $\frac {2}{3}$MRM, sensitivity and specificity. Accompanying ROC-curve plots were also generated. This simulation was repeated for 4—9 cycle-diaries.

All statistical analyses were performed with the statistical software R (v.3.4.0, 2017-04-21) within the RStudio platform; plots were generated with ggplot2 and plotly [19–22].

### Ethics

All data were fully anonymised prior to analysis for this study. At the time of data collection (1996—1998) consent was not required for surveillance studies [4].

## Results

### Descriptive statistics

A total of 46 (27.9%) diaries were excluded: 38 did not contain three consecutive menstrual cycles; 8 contained menstrual cycles of atypical duration; leaving 119 diaries eligible for analysis. A total of 15 358 diary days, 541 menstrual bleeds, 2 153 migraine days, and 1 070 migraine attacks were recorded in the retained data. The women recorded an average of 4.5 menstrual cycles (median = 4; range = 3—15). The median of the individual mean cycle lengths was 28.0 days (mean = 28.8, range = 15—84). See also Table 1.

**Table 1:** Descriptive statistics for the migraine diaries in the data set

	Median^*^	(IQR)^*^	Mean^*^
Diary-days observed	103	(83.0—151.5)	129.1
Migraine locked days per 30 days	0.1	(0.0—0.4)	0.3
Age (of the patients in years)	42.0	(35.8—47.0)	41.0
Migraine days	14.0	(10.5—22.0)	18.1
Migraine attacks	7.0	(5.0—10.0)	9.0
Migraine days per 30 days	3.8	(3.1—5.2)	4.3
Migraine attack duration (days) ^*†*,*‡*^	1.0	(1.0—2.0)	1.8
Number of menstrual cycles	4.0	(3.0—5.0)	4.5
Cycle lengths (days) ^*‡*^	28.0	(25.8—30.0)	28.8

^*^The statistics reported (e.g. the means) are taken over the individual patient-headache diaries

^†^I.e. the number of consecutive days of migraine – see also Fig. 3

^‡^The figures reported are the median of the individual medians, and the mean of the individual means

### Comparison of $\frac {2}{3}$MRM and sMM

**Fig. 4:**
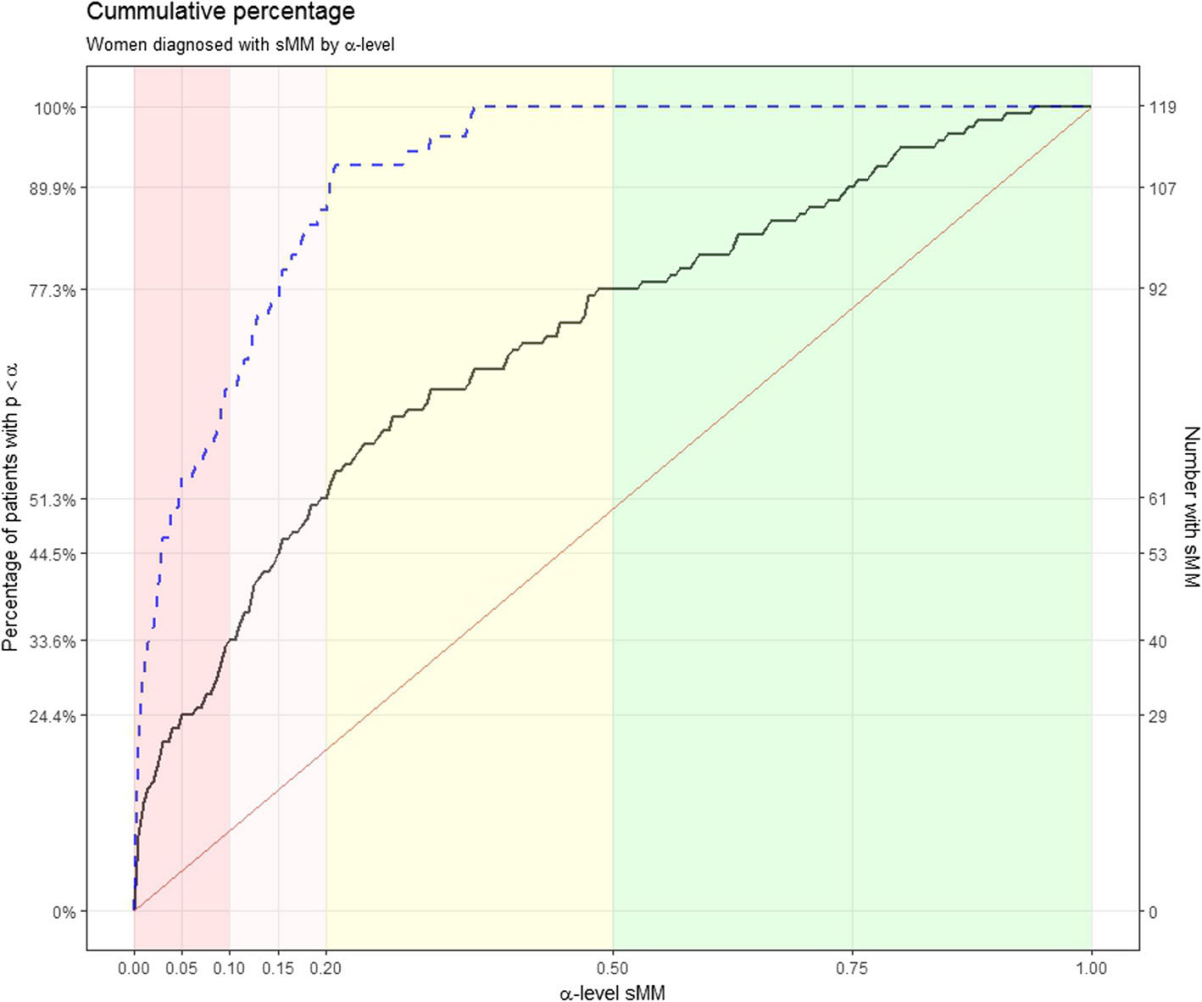
Solid, black curve plots the percentage [number of women] on the left [right] *y*-axis receiving an sMM diagnose by *α*-level on the *x*-axis. The dashed, blue curve plots the percentage of women with a $\frac {2}{3}$MRM diagnosis that receive an sMRM(*α*)-diagnosis. The red line represents the expected distribution, given that all women satisfy the test’s null hypothesis of non-association (i.e. constant *μ*); as such the areas between the solid black curve and the red line can be interpreted as a measure of the aggregated association between menstruation and migraine in the patient population

Among the 119 women, 54 (45.4%) fulfilled the criteria for $\frac {2}{3}$MRM. For sMM the number of women diagnosed depended on the chosen *α*-level (Fig. 4).

**Fig. 5:**
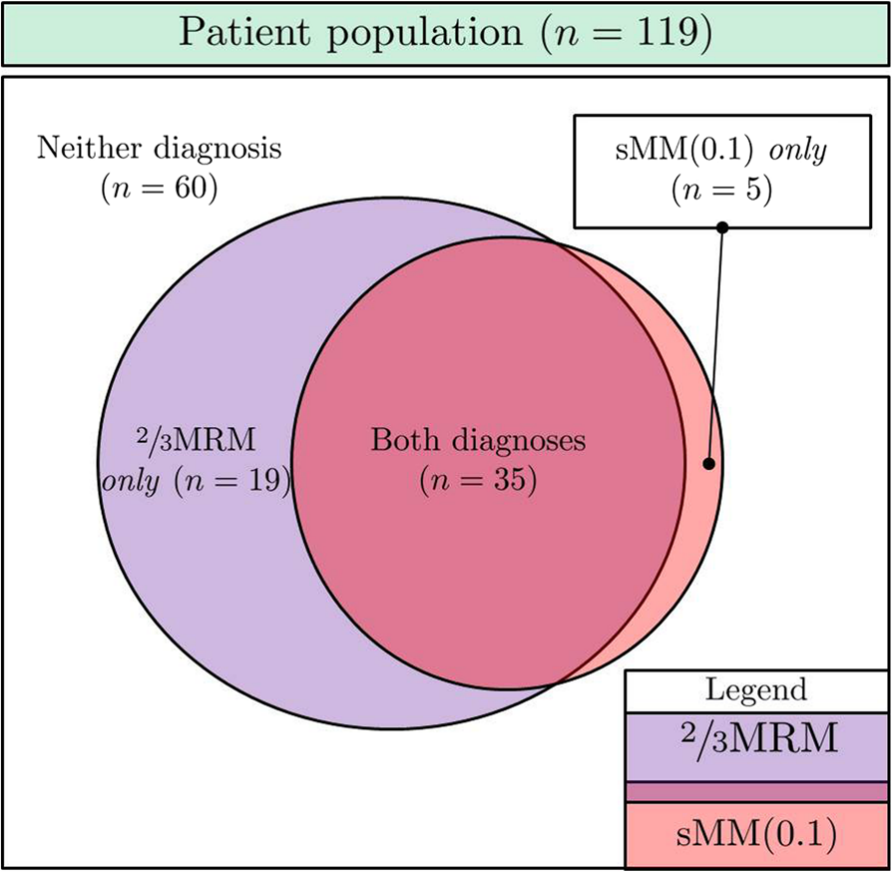
Venn diagram for the patient population classified by $\frac {2}{3}$MRM and sMM(0.1). We observe considerable concordance between the two diagnoses, but also noteworthy discrepancies

We (arbitrarily) set *α*=0.1 for diagnosing sMM in the subsequent analyses comparing those who were diagnosed with either/neither $\frac {2}{3}$MRM and/or sMM; see Fig. 5. This *α*-level seems a reasonable compromise between sensitivity and specificity for MM. However, it is important to note that about 10% of those without an association will then be diagnosed with sMM(0.1): the specificity of sMM equals 1−*α* by construction.

Summary statistics for the diagnose-based subgroups are displayed in Table 2.

**Table 2:** Descriptive statistics and estimated Markov chain model parameters for diagnosis-derived subgroups

		Subgroups – by diagnosis					
		Neither diagnosis	sMM	$\frac {2}{3}$MRM	sMM only	$\frac {2}{3}$MRM only	Both diagnoses
***n***		60	40	54	5	19	35
**Age years**	Mean	40.8	41.8	41.3	40.8	39.9	42.0
	Median	43.0	43.0	41.5	42.0	39.0	44.0
	IQR	35.0—47.0	36—45.5	36.0—47.8	35.0—45.0	33.0—49.0	36.5—46.0
**Diary length (days)**	Mean	130.7	143.9	122.4	181.2	92.6	138.5
	Median	104.5	122.0	94.5	134.0	80.0	116.0
	IQR	86.5—152.0	88.3—162.3	81.3—148.5	109—152	76—97.5	85.5—158.5
**No. of menstrual cycles**	Mean	4.7	5.0	4.3	5.6	3.4	4.9
	Median	4.0	4.0	3.0	5.0	3.0	4.0
	IQR	3.0—5.0	3.0—6.0	3.0—5.0	4.0—5.0	3.0—3.0	3.0—6.0
**Migraine days/30 days**	Mean	4.3	3.9	4.4	3.6	5.3	3.9
	Median	3.6	3.6	4.1	3.6	4.8	3.6
	IQR	3.0—5.3	3.1—4.7	3.1—5.3	3.1—4.6	3.4—7.1	3.1—4.9
**Migraine attacks/30 days**	Mean	2.1	1.9	2.2	1.6	2.6	1.9
	Median	2.0	1.9	2.1	1.6	2.6	2.0
	IQR	1.5—2.6	1.3—2.2	1.7—2.6	1.1—1.9	2.2—3.1	1.4—2.2
**Mean attack length (days)**	Mean	1.7	2.0	1.9	2.1	1.6	2.0
	Median	1.7	1.9	1.7	2.0	1.6	1.9
	IQR	1.3—2.0	1.4—2.4	1.3—2.1	1.7—2.5	1.2—1.8	1.3—2.3
**Model param. est.**							
*μ* ^NM^	Mean	8.4	4.5	6.2	4.8	9.3	4.5
	Median	8.1	4.4	5.6	5.0	8.9	4.4
	IQR	5.8—11.0	2.4—6.1	3.1—8.6	3.0—6.1	7.0—11.9	2.1—6.1
*μ* ^M^	Mean	8.3	23.2	22.5	14.2	19.0	24.4
	Median	7.7	22.9	21.6	13.6	18.2	23.1
	IQR	5.2—11.1	11.8—27.3	17.1—27.1	11.8—13.6	16.0—20.0	20.0—27.3
*Δ* *μ*	Mean	0.0	18.6	16.3	9.4	9.6	20.0
	Median	0.0	18.2	15.5	8.9	9.3	18.6
	IQR	-4.7—4.1	14.2—21.2	10.9—20.2	8.7—9.4	7.4—11.2	15.1—22.1
*μ*	Mean	8.3	7.2	8.6	6.1	11.0	7.3
	Median	7.6	7.1	8.1	6.3	10.6	7.4
	IQR	5.7—10.5	5.1—8.3	6.1—10.6	4.2—7.6	8.8—13.0	5.1—8.7

All women *N*=119

Women who fulfilled the $\frac {2}{3}$MRM-criteria exclusively – i.e. $\frac {2}{3}$MRM but *not* sMM(0.1) – presented with fewer recorded cycles, and elevated overall migraine frequencies; i.e. the typical candidate for being a false positive. Conversely, the five women who fulfilled the sMM(0.1) criteria exclusively had longer observational lengths, but lower migraine frequency. The group of sMM-exclusive women all had sMM *p*-values in the range 0.05—0.10, and represent roughly the expected count of false positives given $\frac {2}{3}$MRM as the ‘gold standard’. If, conversely sMM(0.1) is held as a ‘gold standard’, this suggests that $\frac {2}{3}$MRM is quite sensitive, but unacceptably unspecific.

**Fig. 6:**
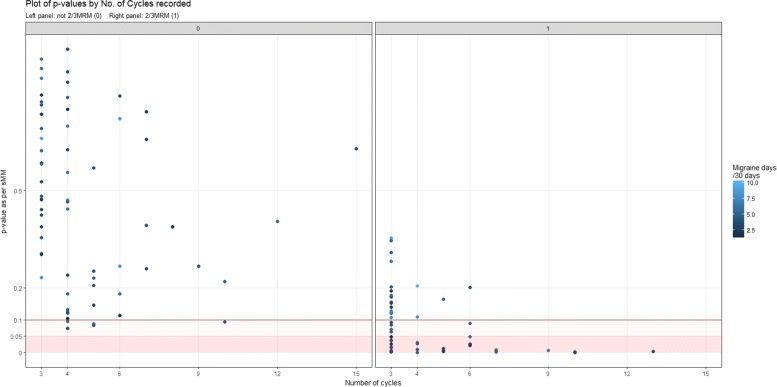
Plot of the individual sMM *p*-values (*y*-axis) against the number of cycles recorded (*x*-axis). The left panel contains the women without a $\frac {2}{3}$MRM diagnosis, the right those with a $\frac {2}{3}$MRM diagnosis. The lighter the dot is, the lower is her estimated migraine frequency. We see that amongst the women with an $\frac {2}{3}$MRM diagnosis, the *p*-values appear to be low when many cycles were observed, while the *p*-values for women with few recorded cycles are more dispersed (along the *y*-axis). The women with relatively low *p*-values in the left panel represent women with low migraine frequency, and more cycles recorded

Figure 6 displays this relationship graphically, and also visualises the differences between the two methods with respect to migraine frequency and the number of cycles recorded.

In an ad hoc sub analysis, we also computed summary statistics for the 27 women with six or more recorded cycles, under the rationale that more information ought to yield more trustworthy estimates. The general trends remained; see Table 3.

**Table 3:** Descriptive statistics for diagnosis-derived subgroups

		Subgroups – by diagnosis		
		Neither diagnosis	Either diagnosis	Both Diagnoses
***n***		14	13	11
**Age years**	Mean	40.5	40.7	41.7
	Median	41.5	38.0	40.5
	IQR	35—51	36.0—45.8	36.3—47.3
**Diary length (days)**	Mean	228.4	229.8	221.9
	Median 209.5	192.0	192.0	
	IQR	182.2—239.0	162.0—293.0	162.5—289.5
**No. of menstrual cycles**	Mean	7.0	7.9	7.9
	Median 8.0	7.0	7.0	
	IQR	6.0—8.8	6.0—10.0	6.0—9.5
**Migraine days/ 30 days**	Mean	4.2	4.2	4.3
	Median 3.8	3.6	4.4	
	IQR	2.7—5.2	3.4—5.3	3.2—5.4
**Migraine attacks/ 30 days**	Mean	2.1	2.0	1.9
	Median	1.9	2.0	2.0
	IQR	1.5—2.6	1.4—2.4	1.4—2.2
**Mean attack length (days)**	Mean	1.9	2.1	2.2
	Median	1.3	1.7	1.9
	IQR	1.3—1.9	1.4—2.2	1.4—2.3
**Model param. est.**				
*μ* ^NM^	Mean	8.2	5.5	5.0
	Median	6.9	6.0	5.2
	IQR	5.4—10.1	3.0—7.4	2.8—6.4
*μ* ^M^	Mean	9.1	20.0	20.1
	Median	10.0	18.9	21.4
	IQR	5.7—12.5	16.7—21.7	17.4—22.9
*Δ* *μ*	Mean	0.9	14.4	16.0
	Median	1.3	15.1	15.1
	IQR	-2.0—3.7	12.2—16.2	13.6—17.4
*μ*	Mean	8.3	7.8	7.5
	Median	7.5	8.1	7.9
	IQR	5.6—10.0	5.6—9.0	5.4—8.6

Patients with 6+ cycles recorded. *n*=27 (22.7%)

### Sensitivity–specificity simulation and criteria performance

**Fig. 7:**
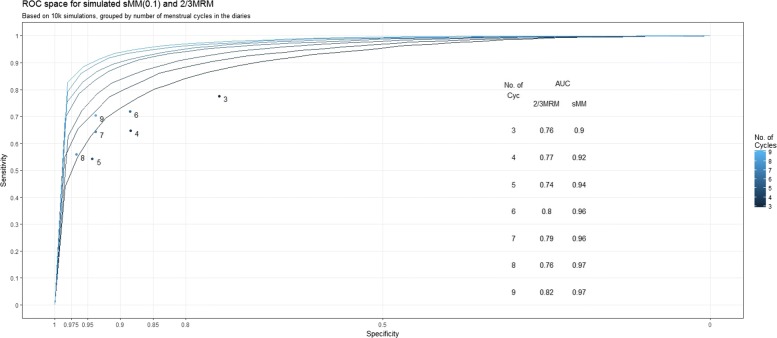
ROC curve plot with AUC scores. Lighter color represents higher number of menstrual windows – i.e. longer headache diaries – in the underlying simulation. Each of the 2×7 (sensitivity and specificity for 3,…,9 cycles) simulations ran on 10 000 diaries

The results of the simulation analyses are contained in Fig. 7. As expected, both methods display increased performance monotonically in the number of cycles observed in the underlying simulation, reflected in an increasing AUC value.

We note that the sMM is superior across the simulations of varying number of menstrual windows.

Strikingly, the $\frac {2}{3}$MRM-diagnosis *loses* sensitivity when the number of observed menstrual windows is increased until the number of cycles reaches the next multiple of three. For the series of simulations involving three, four, and five cycles, we observe an increasing specificity of $\frac {2}{3}$MRM, but an accompanying *drop* in sensitivity, resulting in an overall deterioration as measured by the AUC-value. For six cycles, the AUC-value increases, followed by a similar pattern through seven- and eight-cycle simulations, before the AUC again is increased for the nine-cycle simulation. Furthermore, the *maximal sensitivity* is observed for 3 cycles, revealing this criterion’s inability to convert the additional information into sensitivity for MM.

The sMM, on the other hand, shows the expected monotonic gain in accuracy with increasing information.

## Discussion

We have presented a novel statistical criterion sMM for diagnosing MM in women: a statistically more robust version of previously proposed probability criterion, [8, 10] which is inappropriate given the empirically observed clustering of migraine days [13].

To remedy this we have developed a methodology for quantifying the probability that a woman’s migraine pattern is associated with her menstrual cycle based on (i) a simple model for the progression of migraine attacks (the Markov chain model), and (ii) standard statistical hypothesis tests (Fisher’s exact test). This method improves on previously suggested criteria by being more accurate (fewer false positives and fewer false negatives was shown in the simulation analysis) and more robust (no dubious assumptions like non-clustering of migraine days). We also saw that the sMM identifies most of the women identified by the ICHD’s $\frac {2}{3}$MRM criterion, but is more restrictive; in particular with regard to women with relatively elevated number of migraine days per 30 days. This might mean that the $\frac {2}{3}$MRM criterion yields unacceptably many false positives. We also saw that sMM was able to establish association for a few women that did not satisfy the $\frac {2}{3}$MRM criterion, which highlight that ‘$\frac {2}{3}$’ might be arbitrary.

### Main findings

We found that women with shorter migraine diaries – in particular those that contained fewer 5-day menstrual windows – paired with increased overall migraine frequency, appeared more likely to be diagnosed with $\frac {2}{3}$MRM than sMM: in about $\frac {1}{3}$ of those fulfilling the $\frac {2}{3}$-criterion, the association between migraine and menstruation was weak or even absent as measured by sMM(0.1), suggesting that the current criteria are quite unspecific.

The ICHD $\frac {2}{3}$-criterion is also ambiguous particularly when considering extended diary data over a number of menstrual cycles; some women may fulfil the diagnostic criteria for some periods during the total period of observation, but not during other periods. For example, a woman with four cycles and migraine in the first two will not fulfil the criteria because of the 4th cycle. If she had only recorded 3 cycles, she would be diagnosed with MM. Furthermore, a serious deficiency with the $\frac {2}{3}$MRM criterion is the discrete nature of the test, and the arbitrary cut-off ‘two-out-of-three’. As demonstrated in the simulation study, this feature makes the $\frac {2}{3}$-criterion unable to exploit information gained in e.g. four or five cycle diaries; instead there is an implicit trade-off between sensitivity and specificity which is controlled by the number of recorded cycles, rather than the researcher or clinician. It is beyond the scope of this work to investigate this further but these results suggest that one could choose other cut-off values than $\frac {2}{3}$, depending on the number of cycles recorded, to partially ameliorate this situation.

### Why do we need an alternative diagnostic tool?

Menstrual migraine is still a disorder characterized by large knowledge-gaps. The pathophysiology is incompletely understood and consequently few high-quality studies on medical treatment are available. Most of the current treatment strategies are based on the assumption that oestrogen-withdrawal is a direct or indirect trigger, while other possible mechanisms have received little attention.

To pin down the pathophysiological mechanisms responsible for MM, we need a homogeneous group of women in whom the association between migraine attacks and menstruation is proven, preferably at.05 (or lower) level of significance. In our sample, there were 29 (24%) women with a *p*-value <.05, and 16 (13%) with a <.01-association; these latter also all fulfilled the $\frac {2}{3}$-criterion.

For clinical trials on MM one should be cautious both with respect to sMM and $\frac {2}{3}$MRM: a false association can introduce unwanted noise, while a lower than $\frac {2}{3}$-frequency in menstrual windows could artificially inflate the measured effect of a prophylactic regime. Since the sMM does not take the regularity of attacks into account, it could be necessary to combine the criterion into a ‘$\frac {2}{3}$-s-MM’ criterion if the context of diagnosing women calls for both a certain migraine burden and a high confidence in a true association.

Clinically one would want to treat these women, at least on a watchful waiting basis, with possible further headache diary keeping for obtaining better certainty of association.

The proposed criterion is statistically robust in the sense that if sMM is diagnosed even after only two cycles, the accompanying *p*-value is still valid. A *p*-value of.03 means that there is only a 3% chance that the association observed is spurious. Of course, this is also true for $\frac {2}{3}$MRM: if a woman completing two cycles in her diary had migraine onset during both menstrual windows, then technically she would *already* qualify for $\frac {2}{3}$MRM. This is, incidentally, exactly the problem with the $\frac {2}{3}$-criterion: for such women the information from the third cycle is completely disregarded and it is worrisome that even the presence of several non-menstrual attacks during the third cycle, combined with a migraine-free third menstrual window, would not inform the diagnosis. In our data, of the 54 women diagnosed with $\frac {2}{3}$MRM, 5 (9.3%) of the cases were women with only three cycles recorded, and with migraine in exactly the first two cycles. None of these women got an sMM-diagnosis (*p*-values in the range 0.15—0.35).

Indeed, the $\frac {2}{3}$MRM-diagnosis depends more on *μ* – the overall migraine frequency – than on *Δμ*, because an elevated overall migraine frequency is likely to result in an $\frac {2}{3}$MRM-diagnosis regardless of *Δμ*. Furthermore, a non-zero *Δμ* paired with a low *μ*^NM^ is unlikely to be picked up on. The sMM-method, in contrast, is sensitive and specific only for *Δμ*.

We also remark that the sMM is closer in spirit to $\frac {2}{3}$MRM than the PC in the following sense: the sMM and $\frac {2}{3}$MRM criteria focus on migraine onset during the 5-day menstrual window. The PC is sensitive for overlap with the 5-day menstrual window. We believe this is a further reason to encourage the use of sMM over the PC, if a replacement or complementary criterion for the $\frac {2}{3}$MRM is desirable.

### Limitations

This study has some limitations. Firstly, the data set was not large and the method should be tested on a larger data set before full adoption. Secondly, we rely on a migraine model with temporal unit ‘day’. Some might argue that the ‘hour’ is more appropriate. It is, however, straightforward to adapt the Markov chain migraine model from Barra et al. together with the sMM criterion presented here to any temporal unit, given that rich enough data are available so that its parameters can be estimated [13].

The sMM detects women with a statistical association between migraine and menstruation. Moreover, in contrast to the $\frac {2}{3}$MRM, it does not take the regularity of attacks into account. This means that a combination of both methods could be indicated in certain cases, e.g. in clinical trials. The sMM does not directly distinguish between PMM and MRM, although women with PMM will form a subgroup of women with low *p*-values. Whether a distinction between PMM and MRM is necessary within a population with a significant association is questionable.

Paradoxically, the ultimate aim of developing the sMM diagnosis is that it will catalyse its own redundancy. It is developed as a mean to an end; the end being a pathophysiological-based MRM diagnosis. That is, we would like to identify and treat MM without having to resort to statistical analyses, instead relying on objective biomarkers. In order to achieve this, increased statistical accuracy for recognising MM is wanted.

## Conclusions

The current ICHD-criteria for MRM is a useful screening tool but when diagnostic accuracy is a requisite, the more sensitive and specific sMM diagnosis could subsequently be applied to be used to include only those with an sMM diagnosis. For example, studies exploring pathophysiological mechanisms need to ensure that the association between migraine and menstruation is greater than chance. The sMM diagnosis reported here may be used as a supplement to – or as a replacement for – the appendix criteria in the ICHD.

We do not advocate using this methodology without caution, and applying either $\frac {2}{3}$MRM or sMM to individual patients should be guided by sound clinical judgement. However, in a context of selecting a larger group of patients for certain types of clinical trials, the sMM should be considered as an important aid.

## Data Availability

The raw, anonymised headache diaries are available from https://www.researchgate.net/publication/335577175_Headache_Diaries_DATABASE;10.13140/RG.2.2.18517.37608.

## Footnotes

1 Recently, MM *with* aura has been introduced in the ICHD3. However, in this paper we consider MM without aura; the methods can be applied to both subtypes.

2 Available as a pre-print from url.to.be.provided.upon.acceptance, or by contacting the corresponding author.

3 The obvious catch is the reduction of the number of observations, and thus the power of the test: *N*=9 in the original diary, and only *N*=4 for the trimmed diary.

4 Please note that if one wants to include migraine with aura, the identical framework can be used. However, we focus on migraine without aura here.
